# A Cognitive Model Based on Neuromodulated Plasticity

**DOI:** 10.1155/2016/4296356

**Published:** 2016-10-30

**Authors:** Jing Huang, Xiaogang Ruan, Naigong Yu, Qingwu Fan, Jiaming Li, Jianxian Cai

**Affiliations:** ^1^Institute of Artificial Intelligence and Robotics, Beijing University of Technology, Beijing 100124, China; ^2^Pilot College, Beijing University of Technology, Beijing 101101, China

## Abstract

Associative learning, including classical conditioning and operant conditioning, is regarded as the most fundamental type of learning for animals and human beings. Many models have been proposed surrounding classical conditioning or operant conditioning. However, a unified and integrated model to explain the two types of conditioning is much less studied. Here, a model based on neuromodulated synaptic plasticity is presented. The model is bioinspired including multistored memory module and simulated VTA dopaminergic neurons to produce reward signal. The synaptic weights are modified according to the reward signal, which simulates the change of associative strengths in associative learning. The experiment results in real robots prove the suitability and validity of the proposed model.

## 1. Introduction

Associative learning can be divided into two types: classical conditioning and operant conditioning [1]. As a basic type of learning, associative learning has been studied a lot. Many computational models have been presented. Some are about classical conditioning [2–5]. The stimulus in these models is assumed to have a weight to measure how strongly it predicts the reward. The bigger the weight is, the closer the stimulus is to the reward. Others are about operant conditioning and reinforcement learning which originates from the former [6–10]. A universal frame for both kinds of conditioning is much less studied. In the present study, we try to set up a model to regard the two aspects of associative learning as a whole and explain them in a common way.

Although the two categories are distinguished in some aspects (e.g., the reward does not depend on the actions chosen by the animal in classical conditioning while it does in operant conditioning), they still have many common features [11]. Both of them are concerned with how animals find the causal relationship between reward and the corresponding signs, for example, some stimulus or their actions. Meanwhile, both of them describe how stimulus is associated with response. Given a stimulus S, the animal tries a response *R*. In classical conditioning, if S tends to predict the appearance of reward (e.g., food), the connection is strengthened [12]. While in operant conditioning, if the result is positive, the connection between S and *R* is strengthened, otherwise it is weakened [13].

In essence, associative learning is not a prerogative of human being. Many researches have suggested that even organisms with rather simple neural systems can have such abilities and establish the association between stimulus and response in classical [14] or operant conditioning [15] way. These findings indicate that relatively simple neural network can have the function of associative learning.

At macroscopic level, associative learning is a process during which human beings and animals discover relationships between stimuli, actions, and outcomes. However, at neural level, associative learning is related to synapses' ability to change their strength in signal transmission, which is called synaptic plasticity.

Synaptic plasticity is considered as a prime mechanism for learning and memory. Such idea is firstly studied by Hebb [16] and then gathers a broad consensus among researchers [17–19]. These studies revealed that there is an important link between local plasticity and macrolevel behavioral learning [20]. The synaptic changes of particular pathways in sensorimotor system could lead to the behavioral changes. Meanwhile, multiple researches suggest that synaptic plasticity is often affected by neuromodulators like dopamine [21–25]. Neuromodulation may involve associative learning and work as a type of synaptic gating mechanism. Therefore, synaptic plasticity modulated by neuromodulators is considered to play an important role in conditioning behavior learning [20]. Driven by these findings, we try to construct a model based on synaptic plasticity with neural modulation and apply it to explain associative learning. Here, the synaptic plasticity is artificial and represented by changing the network's connective weights according to learning mechanism.

Another problem is how to represent the weights. According to Yang et al.'s research [26, 27], neurons in the lateral intraparietal area (LIP) may involve simple decision-making, which is similar to action selection in associative learning. Such decision-making may be done in the form of a log likelihood ratio (log⁡LR) in the neural system. Pfeiffer et al. adopted the conclusion and presented a brief frame for neural modulated plasticity [28, 29].

Inspired by the researches above, we present a cognitive model based on modulated synaptic plasticity. The focus of this study is to apply the model to explain the two kinds of associative learning in a unified way. Moreover, as memory plays a fundamental and important role in learning and cognition [30], we add memory module in our model. To find out how memory works in high-level cognitive activities, a number of computational models for memory have been proposed. Many of them focus on two challenging problems: defining the nature of working memory storage and the relationship between working memory and long-term memory. The representative work includes levels of processing [31], parallel-distributed processing [32], models involving hippocampal area of human brain [33], and information processing [34]. Considering the universality, popularity, and influence in history, we adopt information processing model in our work.

This paper is organized as follows. In Section 2, we explain the architecture of the model. In Section 3, we present the working algorithm for the model. In Section 4, we analyze the convergence of the learning mechanism. In Section 5, we reproduce both classical conditioning experiment and operant conditioning experiment in real robots. We also introduce the details about the experiment settings and the structure of the networks and analyze the results of the experiments in this section. The paper ends with concluding remarks in Section 6.

## 2. The Architecture of the Model

**Figure 1:**
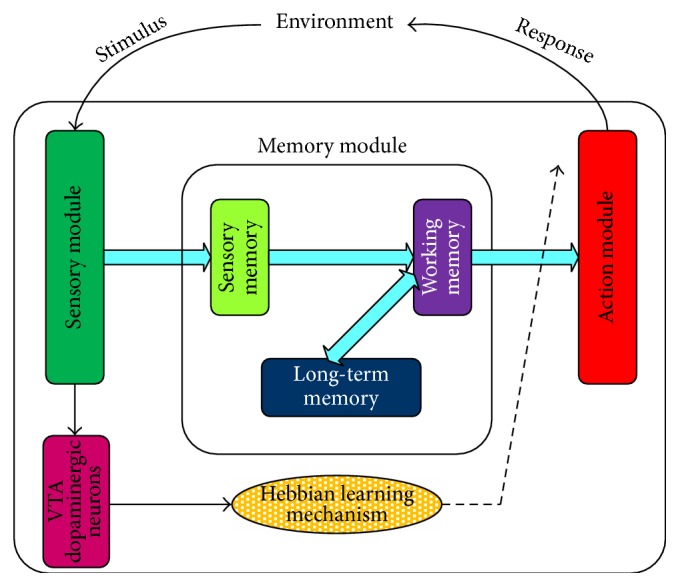
The architecture of the cognitive model. The model includes 3 main modules: sensory module, action module, and memory module. Thus, the interactions between agents and environment, that is, stimulus and response, are transferred as the information flows from sensory module to action module, signifying the sensorimotor system. Besides, VTA dopaminergic neurons and learning mechanism work together as the learning system to modify the synaptic weights between working memory and action module. All the above, the agent and the environment, compose a close-loop system.

The architecture of our model is shown in Figure 1. The relationship between stimulus and response is modeled as the mapping from perception to motor, represented by the information stream from sensory module to action module. Meanwhile, as mentioned above, memory plays an important role in cognition. Therefore, 3-layer memory module is added in the model. Learning mechanism here refers to the rule of changing the synaptic weights between working memory and action module, which makes the model self-learning and self-organized. VTA dopaminergic neurons are also simulated here to represent the neuromodulation and to produce the reward signal for the learning mechanism.

### 2.1. Sensory Module

The sensory module represents sensors in animals or robots. It collects and receives stimulus from environment, which will soon be transmitted to sensory memory. Its output also provides the unit of VTA dopaminergic neurons, helping judge whether there is a reward or not.

### 2.2. Memory Module

Memory is important in cognition. Here, we adopt the three-layer architecture to describe the memory module. They are sensory memory, working memory, and long-term memory.

*Sensory Memory*. Sensory memory stores sensory information from sensory module just long enough to transfer it to next memory unit: working memory. Its function is to provide a snapshot of agents' overall sensory experience and retain the impressions after the original stimulus has stopped.

*Working Memory*. Working memory, the second layer of the multistore memory model, receives the output from sensory memory. It plays the role not only of a bridge between sensory memory and long-term memory, but also of the key for learning and memory. Working memory processes the information from sensory memory to make it easy to handle, that is, memory coding, and delivers it to action module.

Meanwhile, working memory records the statistics of each action with reward or without reward, which offers data for learning mechanism. All results for each action selection, that is, the numbers of times of reward or no reward for each serial action, will be saved in working memory. For instance, suppose there is an action chain: *a*_1_ *a*_2_ ⋯ *a*_*n*_ (*n* ≥ 1), in which *a*_1_ is the first action while *a*_*n*_ is the last one. *R*_1,2,…,*n*_ records the number of times of reward after the action chain is selected, while ${\bar{R}}_{1,2,\ldots ,n}$ represents the number of times of no reward. Both *R*_1,2,…,*n*_ and ${\bar{R}}_{1,2,\ldots ,n}$ will be saved in working memory.

Finally, working memory communicates with long-term memory for accumulating and taking advantage of learning experience. Every time when learning starts, it loads the last time learning result from long-term memory and stores new learning result at the end of learning.

*Long-Term Memory*. Long-term memory along with working memory and sensory memory constitutes the complete memory mechanism. The main function of long-term memory is to save the learning result, which represents the experience accumulated through the interaction of agents with the environment. Every time when learning starts, the long-term memory is retrieved and loaded to working memory for new learning. When learning ends, the result is saved in long-term memory.

### 2.3. Action Module

**Figure 2:**
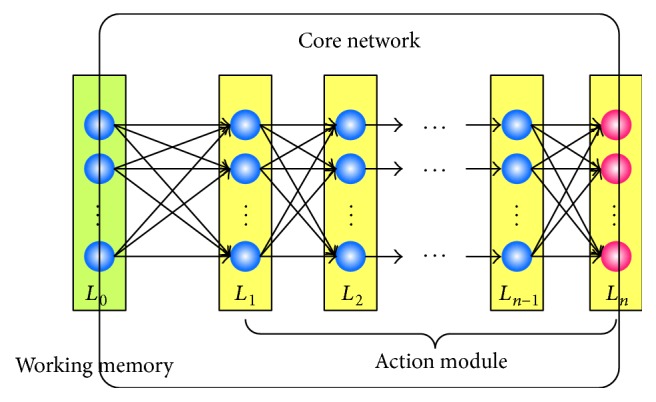
The structure of the core network. Working memory and action module are connected in a full connective way; that is, every neuron in the former layer is connected to all neurons in the next layer and so on. Neurons in working memory are ADALINE neuron [35] whose threshold value is 0, shown as blue ones in the figure. All the neurons of the action module except the ones in the last layer are the same. The neurons in last layer are perceptron neurons [36] whose output is discrete and easy to use, shown as red ones in figure.

Action module represents effector or neurons related to actions. Its input comes from working memory, while its output represents the expression of actions. The action module along with working memory, especially the connections between them, is the core of the whole model. The structure of the core network is shown in Figure 2.

As illustrated in Figure 2, action module consists of multiple layers of neurons. Each layer represents one time of action selection while each neuron represents one action. In fact, the network in action module indicates the action chains learned.

The actions will be chosen in winner-take-all way. In other words, the action with the biggest connective weight will be chosen.

Suppose at time *t* the input vector for a neuron in action module is **x**(**t**) = (*x*_1_(*t*), *x*_2_(*t*),…, *x*_*n*_(*t*))^T^, and the corresponding weight vector is **w** = (*w*_1_, *w*_2_,…, *w*_*n*_)^T^. If the neuron is not in the last layer, its output is calculated as follows:

(1) $$\begin{array}{c} o\left(\;t\;\right)=\overset{n}{\underset{i=1}{\sum }}w_{i}x_{i}\left(\;t\;\right)=w^{T}x\left(\;t\;\right). \end{array}$$

Otherwise, its output is calculated as follows:

(2) $$\begin{array}{c} o\left(\;t\;\right)=\left\{\begin{array}{cc} 0 & u\left(t\right)<b\\ 1 & u\left(t\right)\geq b. \end{array}\right. \end{array}$$

The symbol *b* in formula (2) signifies the threshold value, or bias, of the neuron. And *u*(*t*) = ∑_*i*=1_^*n*^ *w*_*i*_ *x*_*i*_(*t*) = **w** ^T^ **x**(**t**).

### 2.4. VTA Dopaminergic Neurons

Reward signal is a crucial factor in associative learning. As mentioned above, many evidences show that neuromodulation plays an important role in mediating the reward signal. The ventral tegmental area (VTA) in midbrain area is believed to be the neural substrate of such modulation [37].

VTA is one of the most important dopaminergic areas. The best-developed current theory of dopaminergic function is the “reward prediction error” hypothesis that dopamine encodes the difference between actual and predicted rewards [38, 39]. The magnitude of phasic dopamine-neuron bursts quantitatively represents positive prediction errors [40].

The idea can be expressed in the following formula where *δ* represents the dopamine signal, *R* represents the actual reward, *R*′ represents the predicted reward, and *k* is the positive coefficient:

(3) $$\begin{array}{c} \delta =k\times \left(\;R-R^{\prime }\;\right). \end{array}$$

In this work, agents are supposed to have no expectations about reward, that is, *R*′ = 0. Thus, the dopamine signal can be regarded as proportion to actual reward; that is,

(4) $$\begin{array}{c} \delta =k\times R. \end{array}$$

To calculate the dopamine signal, we introduce the concept* ideal degree* (ID) in this model. It is a numeric value decided by specific applications and describes how ideal the status agents perceive is. The bigger the ideal degree is, the better the corresponding status is. We assume that the ideal degree will increase if agents get reward and it will decrease if not. Based on such an assumption, a reward is regarded as the function of ideal degree. Suppose at present time *t* that the status perceived is *s*, and its ideal degree is ID(*s*). After the serial action *a* is executed, the status transfers to *s*′, whose ideal degree is ID(*s*′). Then, the reward function *R* can be defined as follows:

(5) $$\begin{array}{c} R=\frac{2}{1+e^{-\Delta ID}}-1, \end{array}$$

where ΔID = ID(*s*′) − ID(*s*). Then, we can calculate the dopamine signal according to formula (4) and (5).

As formula (3) shows, *R* is a sigmoid function with the value domain (−1,1). The function is on the symmetry of origin and monotonically increasing. When ΔID > 0, that is, ID(*s*′) > ID(*s*), then *R* > 0, indicating that the ideal degree increases and agents get reward by selecting the action chain. Moreover, since it is monotonically increasing, the more ideal degree increases, the bigger *R* becomes. The extreme case is that *R* will approach 1 in case ΔID approaches infinite. On the contrary, if ΔID < 0, then *R* < 0, indicating that agents have not been rewarded and the status is worsening. The more the ideal degree decreases, the less *R* becomes. When ΔID → −*∞*, *R* → −1. A special case is ΔID = 0, that is, ID(*s*′) = ID(*s*); then *R* = 0. The case is regarded as unrewarded.

## 3. Working Algorithm of the Model

Working algorithm describes how the model works and the input is transformed to the output. We introduce a new concept system entropy as a measure of convergence in the working algorithm.* System entropy* (denoted as SE), like entropy in information theory, is calculated as follows:

(6) $$\begin{array}{c} SE=-\overset{m_{1}}{\underset{i=1}{\sum }}p_{i}\log p_{i}, \end{array}$$

where *p*_*i*_ represents the probability of the selected action *a*_*i*_.

Obviously, system entropy signifies the degree of self-organization. The less it is, the higher the degree of self-organization is. When system entropy approaches its minimum, the model or the working algorithm has converged. We use SE or the learning times as the ending condition for the system.

The whole algorithm is as shown below.

**Step 1 (initialization).**

Retrieve long-term memory and load its content to working memory.

Set *nr*_*i*_ = 0 and ${\bar{nr}}_{i}=0$ (*i* = 1,2,…, *m*_1_), where *nr*_*i*_ represents the number of times of being rewarded for action *a*_*i*_ and ${\bar{nr}}_{i}$ represents the number of times of not being rewarded.

Set the connective weights between neurons in working memory and action module *w*_*ji*_ = 0 (*j* = 1,2,…, *m*_0_, *i* = 1,2,…, *m*_1_,  *m*_0_ and *m*_1_ are, resp., the number of neurons in working memory and action module).

Calculate the* system entropy* according to formula (6) where *p*_*i*_ = 1/*m*_1_; that is, agents select action randomly at the beginning.

**Step 2 (select action in WTA (winner-take-all) way).**

Choose the action *a*_*i*_ with the maximum corresponding weight.

Update the number of being selected for the action *a*_*i*_:  *N*_*i*_ = *N*_*i*_ + 1.

Update the probability of each selected action as follows:

(7) $$\begin{array}{c} p_{i}=\frac{N_{i}}{\sum_{j=1}^{m_{1}}N_{j}}. \end{array}$$

Update the system entropy SE.

**Step 3 (observe the response from the environment, judge whether the action is rewarded, and then get the output of VTA dopaminegic neurons).**

Get the new perceived information through sensory module.

Update the representation of the sensory information in sensory memory and working memory.

Calculate the dopamine signal according to formula (4) and (5).

For each action *a*_*i*_ of the action chain being learned, update *nr*_*i*_ and ${\bar{nr}}_{i}$ as follows.

If action *a*_*i*_ results in reward,

(8) $$\begin{array}{c} nr_{i}=nr_{i}+1. \end{array}$$

Otherwise,

(9) $$\begin{array}{c} {\bar{nr}}_{i}={\bar{nr}}_{i}+1. \end{array}$$

**Step 4 (adjust the weights related).**

For each weight *w*_*ji*_ related to the action sequence being learned, the following happens.

If corresponding action *a*_*i*_ results in reward,

(10) wjiln⁡nri+1nr¯i=ln⁡nrinr¯i1+1nri=wji+ln⁡1+1+e−wjinri+nr¯i.

Otherwise,

(11) wjiln⁡nrinr¯i+1=−ln⁡nr¯inri1+1nr¯i=wji−ln⁡1+1+ewjinri+nr¯i.

**Step 5 (judge whether the action module should be changed).**

If reward has not been observed after given times learning, a layer will be added in action module, signifying the action chain should be more complicated. The number of the neurons in the new layer is the number of the actions allowed to be selected.

**Step 6 (judge whether the learning has come to the end).**

If SE is low enough or the learning times have exceeded the maximum limit, then end the algorithm; otherwise, get back to Step 2.

## 4. Convergence Analysis of the Learning Mechanism

The learning mechanism in the model is shown in formula (10) and (11). We modify them before analysis in a briefer form.

Let *t* represent the learning times and *A* represent the action sequence to be learned, in which *a*_*i*_ is an action (*i* = 1,2,…, *n*). When *t* → *∞*, $(nr_{i}+{\bar{nr}}_{i})\to \infty$, then, $\left(1+e^{w_{ji}}\right)/\left(nr_{i}+{\bar{nr}}_{i}\right)\to 0$, $\left(1+e^{-w_{ji}}\right)/\left(nr_{i}+{\bar{nr}}_{i}\right)\to 0$. According to* L'Hospital rule*, ln⁡(1 + *x*) is the equivalent infinitesimal to *x* when *x* → 0. Therefore, we can get the following formula by such an equivalent substitution:  

(12) $$\begin{array}{c} \Delta w_{ji}=w_{ji}\left(\;t+1\;\right)-w_{ji}\left(\;t\;\right)=\left\{\begin{array}{cc} \ln\left(1+\frac{1+e^{-w_{ji}}}{nr_{i}+{\bar{nr}}_{i}}\right)=\frac{1+e^{-w_{ji}}}{nr_{i}+{\bar{nr}}_{i}}, & if\;\;receiving\;\;reward\\ -\ln\left(1+\frac{1+e^{w_{ji}}}{nr_{i}+{\bar{nr}}_{i}}\right)=-\frac{1+e^{w_{ji}}}{nr_{i}+{\bar{nr}}_{i}}, & if\;\;not\;\;receiving\;\;reward. \end{array}\right. \end{array}$$

Let $\mu =1/\left(nr_{i}+{\bar{nr}}_{i}\right)$; then formula (12) can be transformed into the following equation:

(13) $$\begin{array}{c} \Delta w_{ji}=\left\{\begin{array}{cc} \mu \left(1+e^{-w_{ji}}\right), & if\;\;receiving\;\;reward\\ -\mu \left(1+e^{w_{ji}}\right), & if\;\;not\;\;receiving\;\;reward. \end{array}\right. \end{array}$$

Obviously, *μ* > 0, so it can be regarded as the learning rate.

According to formula (13), when agents receive reward, Δ*w*_*ji*_ = *μ*(1 + *e* ^−*w*_*ji*_^) > 0, so the weight *w*_*ji*_ between layers will increase continuously, which indicates that the correlation between the action sequence *A* and the reward is increasing, too. Thus, the probability of the corresponding actions being selected is also increasing. In short, those synaptic weights related to the actions, that is, more likely to bring reward, will be strengthened so that the agents will more probably choose the actions.

On the contrary, if agents do not receive reward, Δ*w*_*ji*_ < 0, then the weight *w*_*ji*_ between layers will decrease continuously. Therefore, the whole process can be described like the following: if selecting those actions that are less likely to result in reward, the related synaptic weights will decrease. Then, the actions will be less likely chosen.

Another question is whether there is limitation for the change of synaptic weights. In fact, the change of synaptic weights is bounded in our model, which is in accordance with the biological fact and suggests the convergence of the model.

Let *E*(Δ*w*_*ji*_) represent the expected value of Δ*w*_*ji*_. When *t* → *∞*, we can obtain the following formula (14) based on formula (13):

(14) $$\begin{array}{c} E\left(\;\Delta w_{ji}\;\right)=p\cdot \mu \left(\;1+e^{-w_{ji}}\;\right)-q\cdot \mu \left(\;1+e^{w_{ji}}\;\right), \end{array}$$

where *p* represents the probability of being rewarded while *q* represents the probability of not being rewarded. Obviously,

(15) p=nrinri+nr¯i,q=nr¯inri+nr¯i.

Substituting formula (15) into formula (14), we obtain another formula as follows:

(16) EΔwjip·μ1+e−wji−q·μ1+ewji=nrinri+nr¯i·μ·1+nr¯inri−nr¯inri+nr¯i·μ·1+nrinr¯i=μ−μ=0.

Therefore, when *t* → *∞*, *E*(Δ*w*_*ji*_) = 0, which means the synaptic weight *w*_*ji*_ will stop changing, neither increase nor decrease. Therefore, the boundation of weights is proved.

Besides, we can draw the same conclusion by analyzing the self-organization feature of the model. As mentioned above, we use the concept* system entropy* (SE) to describe the feature of self-organization. When SE decreases, the degree of self-organization increases; that is, the model is converging and the change of weights is becoming less.

Suppose there are *n* sequences of actions, among which *A*_*i*_ is the one with reward while other sequences *A*_*j*_ (*j* = 1,2,…, *n*, *j* ≠ *i*) are those without reward. *p*_*i*_ represents the probability of being selected for *A*_*i*_, while *p*_*j*_ is the probability of being selected for other sequence actions *A*_*j*_ (*j* = 1,2,…, *n*, *j* ≠ *i*). Thus, we can get formula (17):

(17) $$\begin{array}{c} 1-p_{i}=1-\frac{N_{i}}{N}=\frac{N-N_{i}}{N}, \end{array}$$

where *N*_*i*_ represents the number of times being selected for *A*_*i*_ and *N* is the total number of times for all action sequences, *N* = ∑_*i*=1_^*n*^ *N*_*i*_.

When *t* → *∞*, *N* → *∞*, as *A*_*i*_ more possibly results in reward, its number of times being selected will increase constantly while others will decrease; that is, *N*_*i*_ → *∞* and *N*_*j*_ → 0 (*j* ≠ *i*).

Thus, (*N* − *N*_*i*_) → 0 when *N* → *∞*. Then, 1 − *p*_*i*_ = (*N* − *N*_*i*_)/*N* → 0; that is, *p*_*i*_ → 1 and *p*_*j*_ → 0.

Therefore, we can get new system entropy as follows:

(18) $$\begin{array}{c} SE=-p_{i}\log p_{i}-\overset{n}{\underset{j=1,j\neq i}{\sum }}p_{j}\log p_{j}=-1*0-0=0. \end{array}$$

Formula (18) illustrates that SE will decrease to the minimum value when *t* → *∞*, which indicates that the system is self-organized.

## 5. Experiments Design and Analysis

To evaluate our model, we reproduce two classic animal experiments in associative learning. The first experiment is Pavlov's dog experiment, which is concerned with classical conditioning, while the other experiment is Thorndike's cat experiment, which is concerned with operant conditioning. We choose the two experiments because of their powerful influence and great fame in associative learning theory. Both experiments are reproduced in real robots, which echoed the embodied cognition.

### 5.1. Classical Conditioning Experiment: Pavlov's Dog Experiment

To study the mechanisms underlying the digestive system in animals, Pavlov and Anrep carried out a series of experiments [12]. In the most famous one, Pavlov and Anrep's dog experiment, he found that if a bell was sounded in very close association with dogs' meal for several times, the dogs learned to associate the bell sound with meal; that is, they would drool even if there is no food available. The phenomenon in the experiment is called classical conditioning, or Pavlovian conditioning. The procedure described above is acquisition of classical conditioning. Meanwhile, Pavlov and Anrep also found that if the dogs acquiring classical conditioning did not get food after the bell sounded for several times, the dogs would gradually forget the association between the bell sound and meal, that is, the extinction of classical conditioning. We reproduce both acquisition and extinction of classical conditioning in our model.

#### 5.1.1. Experiment Design

**Figure 3:**
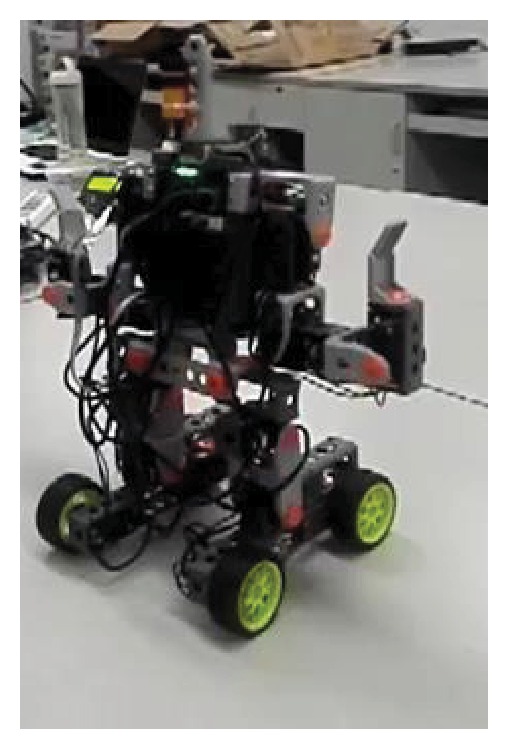
The real robot used in experiment I:* Cogbot I. Cogbot I *is a humanoid robot with 4 wheels. It has 1 infrared sensor and 1 camera. It is also equipped with a small LED screen, which can display the weights in the network.

We carry out the whole experiment (including acquisition phase and extinction phase) in the real robot* Cogbot I*.* Cogbot I* is a humanoid robot with an infrared sensor and a camera, shown in Figure 3.

The infrared sensor and the camera compose the sensory module of the system, in which the infrared sensor represents the dog's ears while the camera represents the dog's eyes. Therefore, an infrared signal, for example, shaking hands near the sensor, represents the sound of bell and works as the conditioned stimulus (CS), while a yellow ball represents food and works as the unconditioned stimulus (US).

Moreover, the registers or buffers in the sensors correspond to the sensory memory. They store the sensory information transiently and provide it to the working memory.

**Figure 4:**
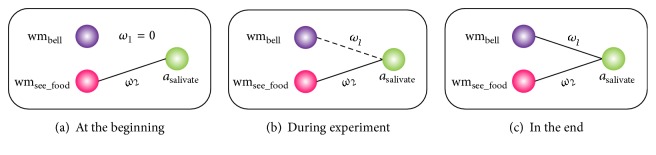
The structure of core network in different phases of acquisition experiment. *ω*_1_ and *ω*_2_, respectively, represent the synaptic weights between the neurons in working memory and action module. At the beginning of the experiment, there is no connection between wm_bell_ and *a*_salivate_, suggesting that the dog will not salivate when it hears the bell sound alone. Thus, *ω*_1_ is 0. Then, it gradually increases, indicating a synaptic connection between wm_bell_ and *a*_salivate_ appears. As it is not big enough to trigger off salivation, the synaptic connection is represented by dash line instead of solid one in (b). At the end of the experiment, *ω*_1_ has increased a lot, so the connective line between two neurons becomes full in (c). During the whole process, *ω*_2_ increases too according to the learning mechanism. However, since the change of *ω*_2_ is not the highlight of the experiment, it is ignored in figure.

Working memory and the action module compose the core network, whose structure is shown in Figure 4. There are 2 neurons in the working memory: one stores the information related to the sound stimulus, denoted as wm_bell_, while the other one stores the information related to the sight stimulus, denoted as wm_see_food_. The outputs of both neurons indicate that the robot has received corresponding stimuli. For example, if the output of wm_bell_ is 1, it suggests that the robot* hears* the sound of bell. On the contrary, if the output of wm_bell_ is 0, it indicates that the robot* does not hear *any sound. In the action module, there is only 1 neuron corresponding to the action salivation, denoted as *a*_salivate_. In order to make the action visible, we use the action of bending back to represent it. Similarly, its output shows whether the dog salivates: 1 means yes and 0 means no.

The long-term memory of the system stores the learning results, mainly the synaptic weights of the core network. Its initial contents are different in acquisition experiment and extinction experiment. For example, in acquisition phase, *ω*_1_ in the long-term memory is initially set to be 0, symbolizing the robot has not associated the sound of the bell with the presentation of food. On the contrary, in extinction phase, the initial value of *ω*_1_ is positive, symbolizing the robot has learned the association. Each time at the beginning of learning, the contents in the long-term memory will be loaded to the working memory.

The reward signal produced by simulated VTA dopaminergic neurons is designed in this way: as food can bring satisfaction to the dog, we think that the dog's statuses will improve if food is presented; that is, the ideal degree of the statuses will increase. Therefore, the reward signal will be positive according to formula (4) and (5). In short, the dog will be rewarded if food is presented. Otherwise, it will not.

In acquisition phase, as the agent gets reward, both of the synaptic weights *ω*_1_ and *ω*_2_ will increase according to formula (13). That is,

(19) $$\begin{array}{c} \omega \left(\;t+1\;\right)=\omega \left(\;t\;\right)+\mu \left(\;1+e^{-\omega \left(t\right)}\;\right). \end{array}$$

On the contrary, in extinction phase, both of the synaptic weights *ω*_1_ and *ω*_2_ will decrease because of no reward according to formula (20):

(20) $$\begin{array}{c} \omega \left(\;t+1\;\right)=\omega \left(\;t\;\right)-\mu \left(\;1+e^{\omega \left(t\right)}\;\right). \end{array}$$

Although both weights change, only the change of *ω*_1_ is observed and recorded in the experiment as it is the key reason for the explanation of the phenomenon.

We use the learning times as the ending condition. In both acquisition and extinction experiments, the robot has to learn 50 times. After that, the experiments come to an end.

#### 5.1.2. Results of Acquisition Experiment

The process of acquisition is the one that the connection between the sound stimulus and the salivation response is being established. The dog did not salivate at the beginning of the experiment when it heard the bell sound alone. Then, it was fed every time along with the sound. After a few times of such trials, the dog would salivate even if it only heard the sound of the bell.

**Figure 5:**
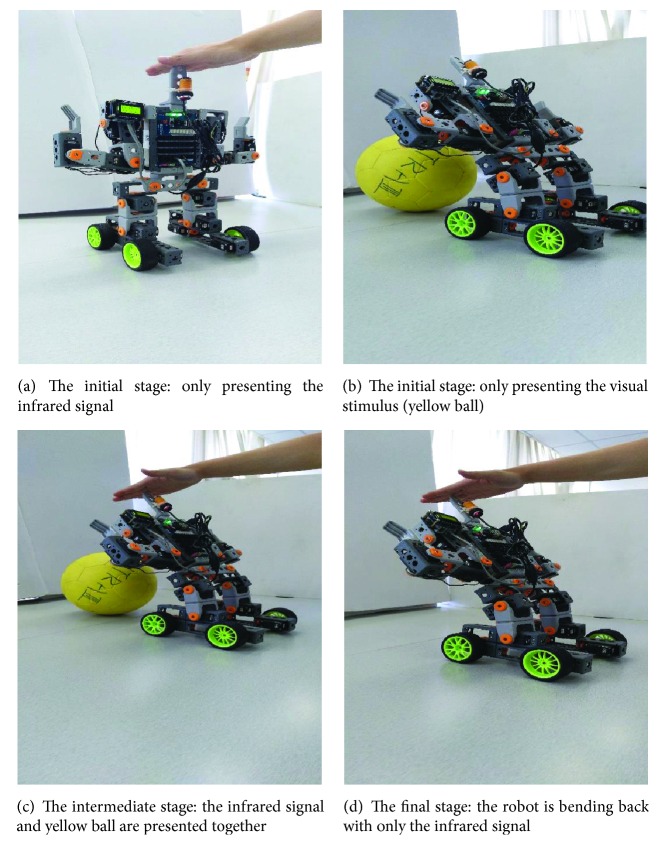
The whole process of the acquisition experiment in* Cogbot I*.

Such process is reproduced in* Cogbot I*, shown in Figure 5. At first, only the infrared signal presents, and the robot has no reaction at all. Then, when the yellow ball and the infrared signal are presented together, the robot bends back since the US is presented. Finally, after a few times of trials, the weight is big enough to excite the corresponding action. Therefore the robot begins to bend back even without the yellow ball.

**Figure 6:**
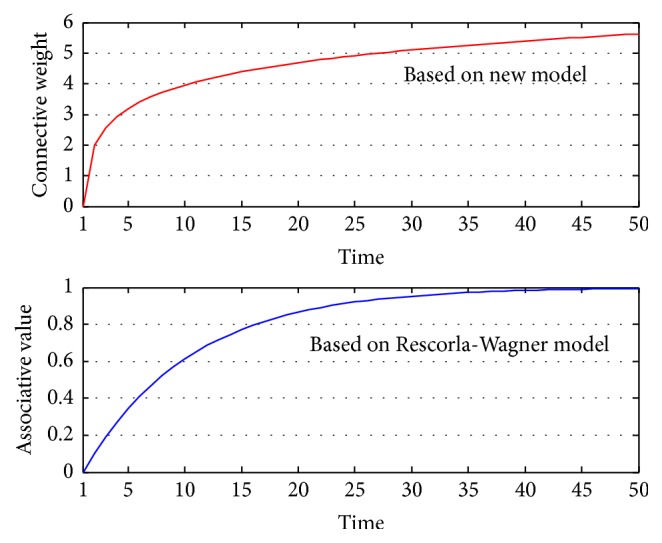
The change of *ω*_1_ in the acquisition experiment and the comparison with Rescorla-Wagner model.

The reason behind the phenomenon lies in the change of the synaptic weight *ω*_1_. As analyzed Section 5.1.1, the weight *ω*_1_ will continuously increase in acquisition experiment until the end of the experiment. The change of *ω*_1_ is recorded and compared with the data of Rescorla-Wagner model [2] (the learning rate is 0.1), shown in Figure 6.

Despite different meanings of these data (our model records connective weight while Rescorla-Wagner model records associative value), the tendencies reflected by the two models are the same. During the process, both connective weight and associative value continuously increase, which indicates the agent has gradually learned to associate CS with the reward and classical conditioning is being acquired.

According to formula (2), only when *ω*_1_ is bigger than the threshold value of the neuron *a*_salivate_, denoted as *b*, can the action neuron be excited. Therefore, the factor which decides whether the action will be executed without the presentation of food is the value of *b*. At the beginning of the experiment, *ω*_1_ is quite small and does not exceed *b* so that the neuron *a*_salivate_ will not be excited and the action will not be executed, either. However, as *ω*_1_ continuously increases, it will exceed *b* after a few times of learning. Then, the action can be executed even without food.

**Figure 7:**
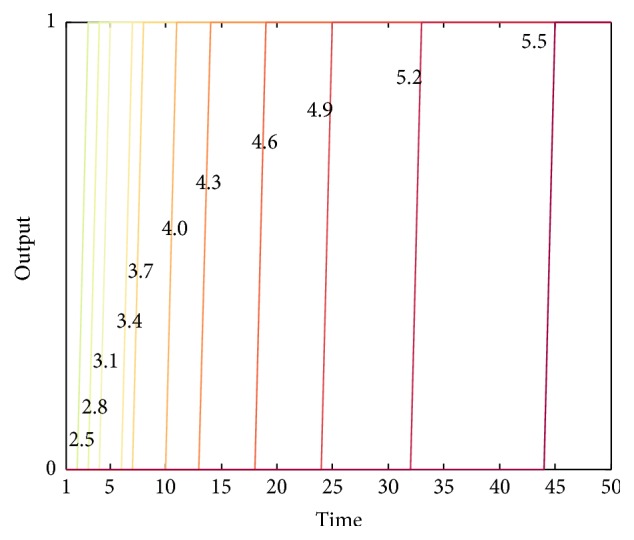
The output of *a*_salivate_ under different threshold values in acquisition experiment. We choose 11 different values in the interval [2.5,5.5]. All the values form an arithmetic progression with common difference 0.3. All *b* values are marked beside the corresponding lines. The color of the lines indicates the values: the darker the color is, the bigger the *b* value is.

We discuss the influence of different threshold values on the output of *a*_salivate_, shown in Figure 7. The figure shows that the less *b* is, the more easily *a*_salivate_ is excited; that is, its output becomes 1. Since the model is proved to converge, the increment of *ω*_1_ is less and less during the experiment. Thus, the gaps between neighboring lines in Figure 7 become wider and wider.

#### 5.1.3. Results of Extinction Experiment

Contrary to acquisition phase, extinction phase is the process during which the association between CS and the conditioned response gradually disappears. In Pavlov's dog experiment, if CS, that is, the bell sound, is presented alone without food repeatedly, the conditioned response, that is, salivation, will be no longer watched.

**Figure 8:**
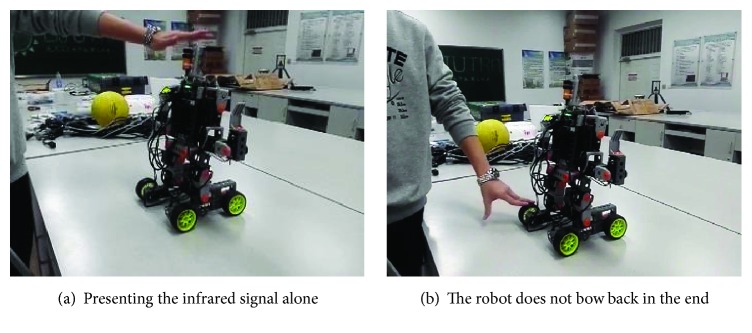
The results of the extinction experiment in real robot* Cogbot I*.

We reproduce it in the robot* Cogbot I*. The extinction experiment is done right after the acquisition experiment. Therefore, the robot at the beginning of the extinction experiment acquires the classical conditioning. Then, we present CS (the infrared signal) alone without US (the visual signal) every time we make experiments. At the end of the experiment, the robot does nothing when CS is presented alone, shown in Figure 8.

The phenomenon in the extinction experiment can also be explained from the point of the comparison between *ω*_1_ and *b*. In the extinction experiment, *ω*_1_ at first is so great that it exceeds *b* and the neuron *a*_salivate_ is excited to execute the conditioned response. However, since there is no food presented during the experiment, the synaptic weight *ω*_1_ decreases gradually according to formula (20), suggesting that the association between CS and the conditioned response is being weakened. At certain moment, *ω*_1_ decreases to be less than *b*. Then, the conditioned response cannot be executed any longer.

**Figure 9:**
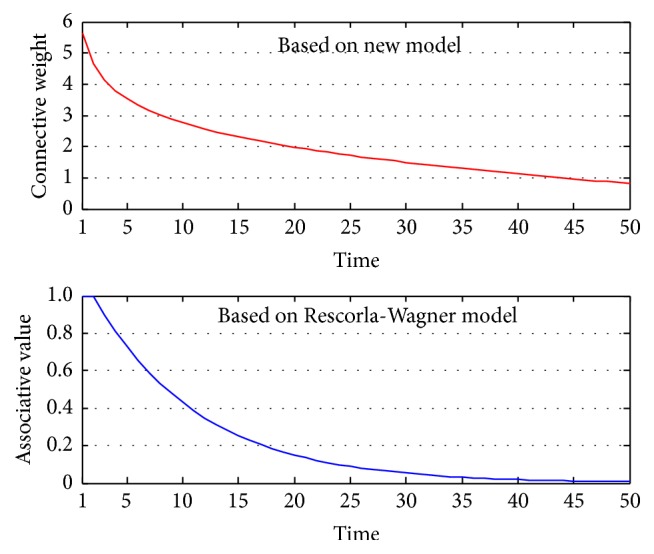
The change of *ω*_1_ in the extinction experiment and the comparison with Rescorla-Wagner model.

We record the change of *ω*_1_ during the experiment and compare it with that of Rescorla-Wagner model, shown in Figure 9. Both the models show a similar process during which the association between CS and the conditioned response is diminishing.

**Figure 10:**
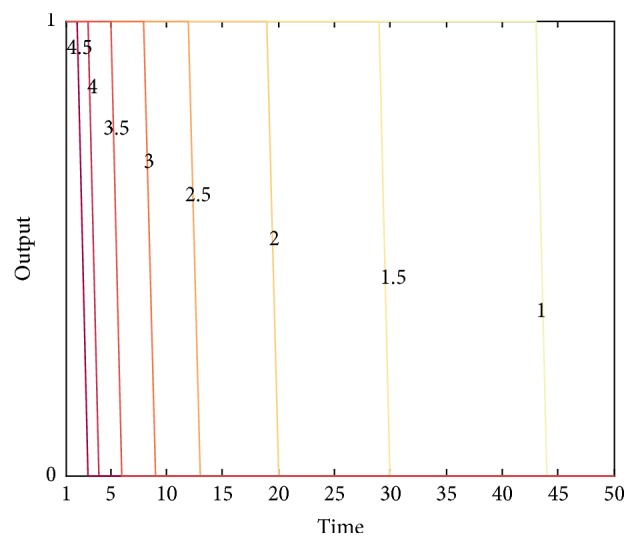
The output of *a*_salivate_ under different threshold values in extinction experiment. All the threshold values are in the interval [1,4.5] with common difference 0.3. We mark all *b* values on the corresponding lines. The color of the lines indicates the values: the darker the color is, the bigger the *b* value is.

We also discuss the influence of different *b* in the experiment.  Figure 10 shows the result. Obviously, the bigger the *b* value is, the faster the extinction procedure is.  Figure 10 still shows a similar phenomenon: the gaps between lines are getting wider and wider, suggesting the decreasing speed of weight *ω*_1_ is slowing down as the experiment continues.

### 5.2. Operant Conditioning Experiment: Thorndike's Cat Experiment

Another type of associative learning is operant conditioning, or instrumental conditioning. Whereas classical conditioning focuses on the association between conditioned stimulus (CS) and conditioned response, operant conditioning involves learning from the consequences of the behavior. Operant conditioning principles presented by Skinner [41] suggest that the behaviors which result in reward tend to be repeated by animals while the behaviors without reward tend to be avoided.

However, Skinner was not the first psychologist to study operant conditioning. Indeed, Skinner's theory on operant conditioning is developed on the ideas of Thorndike. Thorndike formally studied operant conditioning and reward learning back in the late 1800s. He designed and carried out a lot of animal learning experiments, among which the escape experiment of a cat in a puzzle box is the most famous one.

In the experiment, a cat was put in a puzzle box designed by Thorndike. The cat was encouraged to escape to reach a piece of fish placed outside. To go outside the box, it had to firstly press a pedal and then lift the latch of the box. Only when it finished the series of actions in right order could it escape successfully.

**Figure 11:**
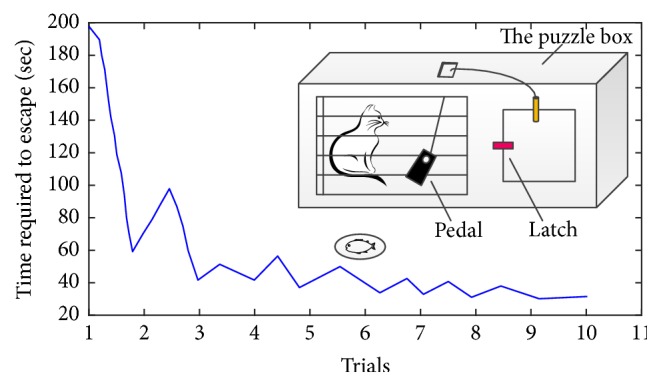
Thorndike's puzzle box and the learning curve in the cat experiment.

Thorndike executed the experiment many times and summarized the results in his learning theory. One is* Law of Effect*, which states that the connections between situations and responses followed by satisfaction are strengthened while the connections with discomfort are weakened. For example, the cat in the experiment would tend to repeat the right series of actions once it found executing such series could bring reward. In fact, the idea of* Law of Effect* is totally in accord with Skinner's operant conditioning theory. Another one is* Law of Exercise*, which states that connections between stimuli and response become strengthened with practice and weakened if practice is not continued. For example, in the cat experiment, Thorndike found that the cat more and more adroitly escaped from the box after it grasped the right method. The time it spent in escaping each time tended to decrease. Thorndike recorded the time and drew a picture, called* learning curve*.  Figure 11 is the learning curve and the puzzle box of the experiment.

Thorndike's cat experiment is not only a good example for both Law of Effect and Law of Exercise, but also a good example for operant conditioning of animals. Therefore, we reproduce the experiment in real robot* Cogbot II* based on our model.

#### 5.2.1. Experiment Design

**Figure 12:**
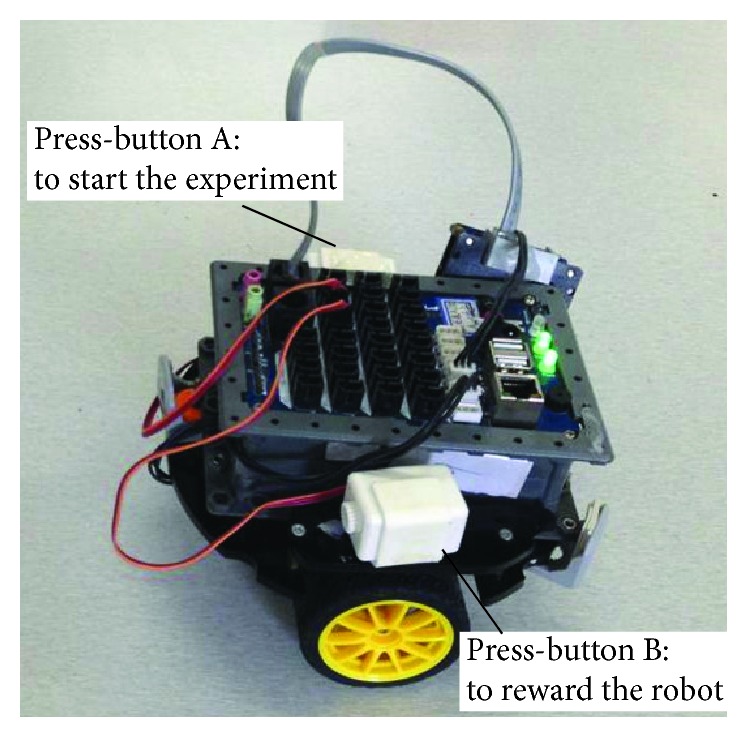
The real robot in the operant conditioning experiment: Cogbot II. Cogbot II is a wheeled robot with 2 press-buttons, among which press-button A is used to start the experiment while press-button B is used to reward the robot.

The operant conditioning experiment is done in the real robot* Cogbot II*, shown in Figure 12.

**Figure 13:**
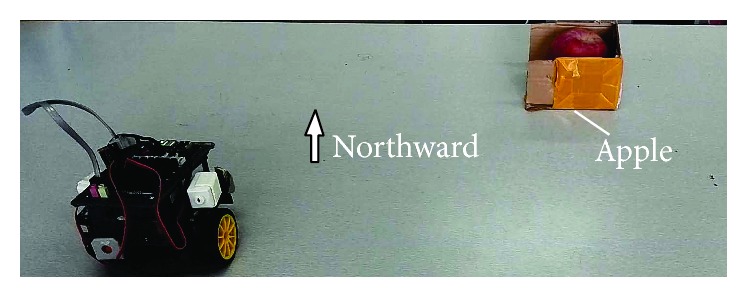
The scene settings of operant conditioning experiment. The aim of the robot is to go northward then eastward to reach the apple.

We simplify the original settings of the cat experiment in the following way: An apple, corresponding to the fish in Thorndike's cat experiment, is put in the north-east of the robot, shown in Figure 13. After we push down press-button A, the experiment starts. The robot can move northward or move eastward. Firstly moving northward then eastward is considered as the only right way to get reward. Each time when the robot chooses a direction, the action will last for 2.4 seconds or 3.5 seconds (if the action includes turning). The speed of the robot and the position of the apple are set just fine to allow the robot to reach the apple in a complete northward-eastward action sequence. If the robot happens to go in the right way, press-button B will be pushed down, representing the robot has got the reward.

In short, the correspondence between the experiment and the original Thorndike's cat experiment may be listed in Table 1.

In the experiment, press-button B composes the sensory module of the system. It is press-button B that the robot depends on to sense the reward. If it is pushed down, it represents that the robot feels the reward. Otherwise, it does not. Similarly, the registers or buffers in the press-button serve as the sensory memory.

The working memory receives information from the sensory memory and codes it in a suitable form for the following operation. In this experiment, there is 1 neuron set in the working memory, denoted as wm_1_. Its output values are 1, 2, and 3, respectively, symbolizing the 3 statuses of the cat, that is, hungry, half-hungry, and full up.

**Figure 14:**
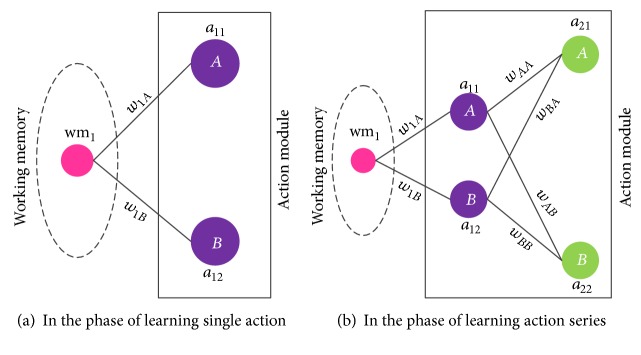
The structure of the core network in the operant conditioning experiment. In the phase of learning single action, there is only 1 layer of neurons in the action module, while there are 2 layers in the module representing the action sequence including 2 actions. The synaptic weights between the working memory and the action module are denoted as *w*_1*A*_ and *w*_1*B*_, while the weights between the 2 layers of the action module are denoted as *w*_*AA*_, *w*_*AB*_, *w*_*BA*_, and *w*_*BB*_, respectively, as shown in the picture.

As mentioned in the second paragraph of this section, each time the robot can choose 2 actions: move northward or eastward. Therefore, 2 neurons in the action module are set to represent them, denoted as *a*_11_ and *a*_12_. Among them, *a*_11_ corresponds to moving northward, and *a*_12_ corresponds to the other. If an action is selected, the corresponding neuron's output is 1; otherwise it is 0. Since the robot learns from single action, there is only 1 layer of neurons in the action module at the beginning of the experiment, shown in Figure 14(a). If the robot is not rewarded all through the single-action-learning phase (set as learning 30 times in this experiment), a new layer consisting of 2 neurons will be added in the action module (shown in Figure 14(b)), which symbolizes that the robot realizes the single action learning does not work and begins to learn more complicated action series. Meanwhile, all neurons in the action module are considered to be excited as long as they receive the outputs of other neurons; that is, the threshold values of the neurons are set to be minus infinity. Every time when the robot executes an action or a sequence of actions, the results for the action or the sequence, that is, the number of times of being rewarded or not, will be saved in the working memory.

Long-term memory stores the results of last time learning. Every time when experiments start, the content in the long-term memory will be loaded to the working memory. When the robot first learns, all of the weights are initialized as 0, indicating that it has no experience to make advantage of.

The reward signal produced by the simulated VTA dopaminergic neurons is computed in the following way.

Firstly, the ideal degree for each state is defined as the value of the corresponding output of wm_1_; that is, the ideal degrees for the state of being hungry, half-hungry, and full are, respectively, 1, 2, and 3.

Each time when the robot chooses an action or a sequence of actions, the states will be transformed in accordance with Table 2. The first line in the table represents all possible action combinations including single action and action series, while the first column represents all the statuses of the cat before executing actions. The entries in the table show the states after executing the actions. For example, the entry on line 1 at column 4 indicates that the hungry cat will be still hungry (wm_1_ = 1) if it chooses the wrong action series *a*_2_ *a*_1_.

Let = 1; then we can get the reward signal *δ* according to formula (4) and (5) based on the state transition and the definition of ideal degree for each state. In general, if the robot chooses the right sequence of actions, the reward signal will be positive; otherwise it will be negative.

In single-action-learning phase, the robot is set to try 30 times. Then, it switches to learn action sequence, which includes 100 trials.

#### 5.2.2. Results and Analysis

As mentioned above, the learning process can be divided into 2 phases: single action learning and action sequence learning. Therefore, experiment results will be listed by stages in this section. The following analysis shows that the results can serve as the evidences of* Law of Effect* and* Law of Exercise*.

**Figure 15:**
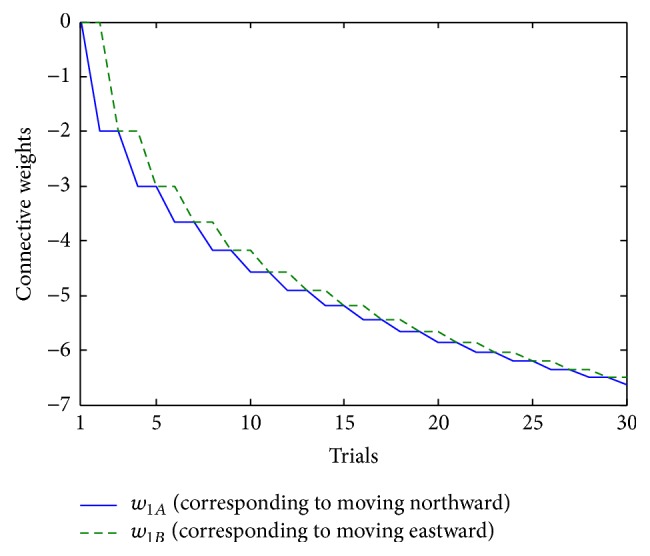
Change of connection weights between neurons in single-action-learning phase.

In single-action-learning phase, no matter which action the robot chooses, it will not get rewarded. Hence, according to the learning mechanism, neither of the two weights related to the actions will increase.  Figure 15 shows how the weights change in the phase.

As shown in Figure 15, since no reward follows the two actions, the weights continually decrease from the original value 0 every time when the related actions are executed.

Indeed, the results in Figure 15 testify one aspect of* Law of Effect*. As the theory states, the connections between the stimulus and the response will be weakened if there is no reward. The synaptic weights between working memory and action module actually symbolize the connections between the stimulus and the response; so what is shown in Figure 15 is completely consistent with the theory.

**Figure 16:**
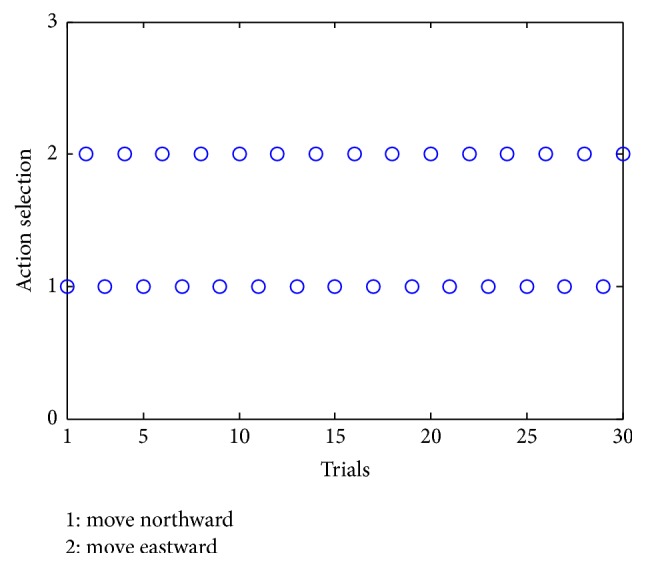
Action selection in single-action-learning phase. 1 represents the fact that the robot chooses to move northward, while 2 represents that it chooses to move eastward.

As a result, such changes influence how the robot chooses actions.  Figure 16 shows the change of the selections in single-action-learning phase. The alternate decreases of the weights make the robot choose the actions in turn during the learning process, as it selects actions in winner-take-all way.

After 30 times of trials, the robot stops learning single action and begins to learn action sequence. The structure of the action module is becoming more complicated and transformed as shown in Figure 14(b).

**Figure 17:**
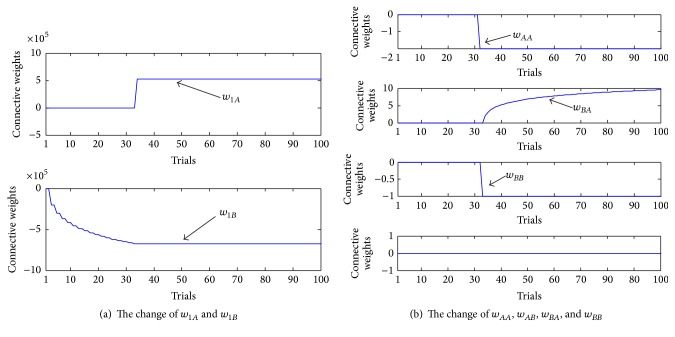
The change of the connective weights of the core network in action-sequence-learning phase.

The change of the synaptic weights is shown in Figure 17. Obviously, only when the robot chooses the right sequence, that is, moving northward first then eastward, can it get reward. Therefore, only the synaptic weights related to this action sequence will increase while other synaptic weights decrease or keep unchanged for the corresponding actions have not been selected all the time.

**Figure 18:**
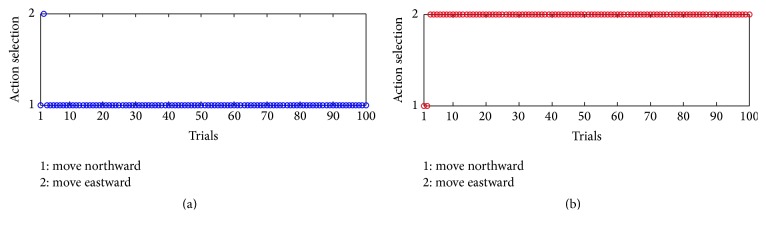
Action selection in serial-action-learning phase. (a) records the change of the first action in the sequence, while (b) is about the change of the second action. The numbers in the figure, 1 or 2, represent the two actions the robot can choose each time.

Similarly, the changes of the weights influence the robot's decision.  Figure 18 illustrates how the robot chooses actions during the phase.

At the beginning of the experiment, since the robot has not learned the association between actions and reward, it makes some wrong decisions. For example, at the first trial, the robot chooses to move northward two times. The second trial is not right, either. However, from the third trial, the robot makes the right choice. As the right action sequence brings about reward and satisfaction, the robot repeatedly chooses the same sequence until the end.

Thus, results in Figures 17 and 18 testify the other aspect of* Law of Effect*, which states that the connections between stimuli and responses will be strengthened if the responses are followed by reward, and those responses or actions will tend to repeat.

**Figure 19:**
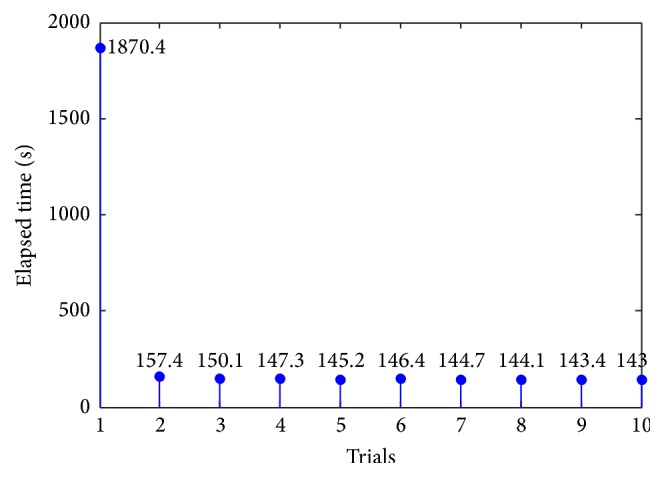
The elapsed time in each experiment.

We also record the spending time of each experiment to validate* Law of Exercise*. When the experiment begins, the timer starts. System entropy, denoted as SE, is calculated during the whole procedure. If SE < 0.3 is observed, the experiment ends and the timer is stopped.  Figure 19 shows the results.

Figure 19 illustrates that the time spent in the experiment is inclined to decrease over time. The robot spent a lot of time in the first trial as it has no experience. However, along with the progress of the experiment, the weights related to the right action sequence were continuously strengthened. Moreover, with the help of the memory mechanism, it had more experience to speed up learning so that it became more and more adept in this task. Such changes are completely consistent with* Law of Exercise*, which states that practice makes perfect.

The whole procedure including single-action-learning phase and action-sequence-learning phase is unsupervised. All the learning behaviors only depend on the interactions between the agent and the environment. Meanwhile, it is also a self-organized procedure in which the structure of the neural network changes autonomously until it converges.

**Figure 20:**
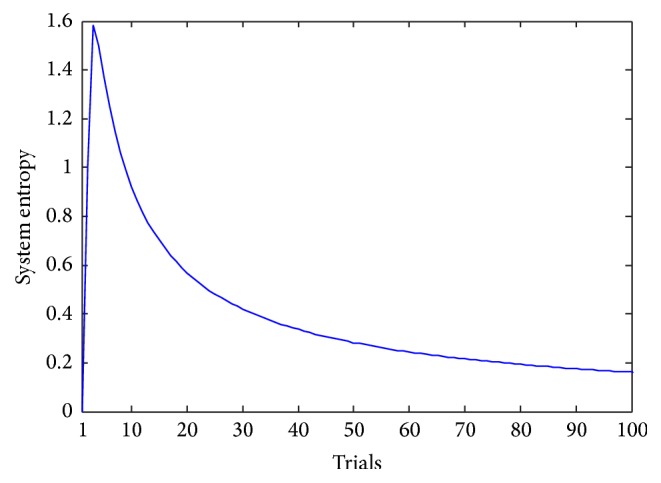
The change of system entropy in action-sequence-learning phase.

As mentioned above, the degree of self-organization can be measured by system entropy.  Figure 20 shows how the system entropy changes during the second phase. The decrease of the system entropy in the phase indicates that the system is changing from disorder to order so that the degree of self-organization increases.

## 6. Conclusion

We have presented a cognitive model based on neuromodulated synaptic plasticity on the issue surrounding associative learning. We apply it to reconstructing two famous conditioning experiments. The results of the experiments in real robots prove the suitability and validity of the proposed cognitive model in different learning tasks. The results also prove the idea that both classical conditioning and operant conditioning are able to be unified in a general frame. The two types of conditioning share a similar neural mechanism and can be unified at the level how stimulus and response connect and how the connections change in the environment.

Moreover, the statistical feature of our model indicates that associative learning is a kind of statistical learning. Some research reports that certain statistical principles like Bayesian rule possibly work in associative learning, in accordance with this study [42].

Finally, this study shows that associative learning is self-organized. As the learning mechanism is unsupervised, the synaptic connections between neurons, or the associative strengths between stimulus and response, change and develop in a self-organized way until new organization forms, signifying the convergence of the model and the emergence of intelligence.

## Figures and Tables

**Table 1:** The correspondence between the operant conditioning experiment and the original cat experiment.

The operant conditioning experiment	The original cat experiment
Robot	Cat
Apple	Fish
Move northward	Press the pedal
Move eastward	Lift the latch

**Table 2:** State transitions in Thorndike's cat experiment.

	*a*_1_	*a*_2_	*a*_1_ *a*_2_	*a*_2_ *a*_1_
wm_1_ = 1	wm_1_ = 1	wm_1_ = 1	wm_1_ = 2	wm_1_ = 1
wm_1_ = 2	wm_1_ = 1	wm_1_ = 1	wm_1_ = 3	wm_1_ = 1
wm_1_ = 3	wm_1_ = 2	wm_1_ = 2	wm_1_ = 3	wm_1_ = 2
